# Does treating Adult ADHD reduce GP mental health workload?

**DOI:** 10.1192/j.eurpsy.2026.12009

**Published:** 2026-07-31

**Authors:** R. N. Bunn, J. Hunter, A. McCauley, J. Gilliland, S. O’Hagan

**Affiliations:** 1 Healthcare in Prison, NHS; 2 Medical School, Queen’s University; 3 GP, University Street Surgery, Belfast, United Kingdom

## Abstract

**Introduction:**

ADHD is a neurodevelopment disorder characterised by inattention, hyperactivity and impulsivity with an estimated prevalence of 3-4% among adults in the UK ^(1)^. First-line pharmacological treatment is with stimulant medications such as methylphenidate or lisdexamfetamine. which are amber listed drugs ^(2)^ and hence must be prescribed according to Shared Care Guidelines ^(3)^. These guidelines list responsibilities for both Psychiatric Specialists and General Practitioners. However, in Northern Ireland, there are currently no commissioned services for diagnosing and treating adults with ADHD in some Trusts, while waiting lists in other Trusts are exceptionally long, causing many patients to seek private ADHD assessments. Increasingly, GPs are not entering into Shared Care Guidelines in cases of Privately Diagnosed ADHD.

**Objectives:**

To determine if treating ADHD in a busy inner city GP practice reduces the workload of GP’S and reduces frequency of missed appointments

**Methods:**

Data was collected from a GP surgery in Belfast which prescribes ADHD medication according to Shared Care Guidelines. Patients were excluded if they had been on ADHD medication for less than 12 months (as of November 2024) or if they had received a diagnosis from childhood. 19 Patients were found to fit the criteria. Data was collected to compare the number of missed appointments pre vs post ADHD diagnosis and treatment, as well as comparing the number of consultations which patients required related to their mental health.

**Results:**

Results from this Audit found that there was no significant difference in the number of appointments which patients missed in the 12 months before ADHD treatment and diagnosis compared to after.

On the other hand, it was found that the number of GP Consultations related to Mental Health almost halved (37 to 19) in the 12 months after ADHD treatment and diagnosis compared to the 12 months before.

Although the sample size was small (n=19), these results indicate that the diagnosis and treatment of patients with ADHD, reduces the burden which they place on Primary Care services. GP’S believe they have a duty of care to the patient and treating ADHD produces a satisfactory outcome for the patient as well as the GP.

Feedback from the GP Practice also included to be mindful that whilst undertaking shared care guidelines for ADHD medication it does have an administration workload attached for the wider practice team ensuring that patients attend reviews before administrating prescriptions to the patients.

**Conclusions:**

Treating ADHD in Adults reduces GP workload and frees up time for other patients.

**Disclosure of Interest:**

None Declared

